# The Patient’s Point of View

**Published:** 1921-12

**Authors:** C. N. Johnson


### THE PATIENT'S POINT OF VIEW

We hear matters discussed in dental meetings affecting the relationship between dentists and their patients and also many remarks in conversation in which the patient's point of view is apparently forgotten or ignored. Men figure out in mathematical precision just how much each hour should net them in practice, just what the overhead is, and just what their "salary" should be. (I hate that word "salary" when applied to a professional practice.) All of this is very laudable and proper so far as it relates to a systemic regulation of one's affairs, and as a means of finding out where there are possible discrepancies in the management of the office. But when men sagely sit with pencil and paper and figure precisely what each hour must bring them willy-nilly, and then start out to apply it rigidly to their practice, they are ignoring one of the fundamental essentials to success. That essential relates to the irrevocable fact that in every transaction between man and man there must be taken into account the point of view of each party to the transaction. And in some of the figuring that has been done by certain dentists, little account has been taken of the point of view of the patient.

It is imperative for every dentist who has any regard for his family or his other obligations in life, to study this matter of the business management of an office practice with some care, so that provision shall be made for those dependent on him, and also for himself in his old age. But to rigidly apply the "pound of flesh" policy to the practice of a profession is not in accord with the best traditions of professional procedure, nor will it tend to create respect or confidence on the part of the public. It has been the chief glory of the professions that they have from time immemorial ministered unto the poor and the needy, and heaven forbid that the day should ever come when they permit their ideas to so change that their activities are dominated solely on the basis of barter and trade. Just as soon as a professional man concentrates on the question of fees, and makes that the dominant note in his dealings with people, just that instant does he lower his professional status and relegate himself to the ranks of commercialism. And there is no more deplorable spectacle than the commercialized professional man.

The point of view of the patient should always be considered in making fees, and no fee should be charged by a dentist that he would not willingly pay himself for the same service under the same conditions. It is true that dentists earn their money by the very hardest kind of work, and they are entitled to adequate remuneration. This is being more and more generally recognized, and people of means are usually willing to pay a good fee. But to make a cut-and-dried rule of a given amount per hour, and then hold patients rigidly to this schedule, is to work a serious hardship on some very excellent people, and to raise the suspicion that dentists are mercenary and are thinking only of the financial aspect of their occupation. Better far to be a poor business man and a good dentist than to be a good business man and a poor dentist; but it is best of all to be a good business man and a good dentist. And there is nothing incompatible in the combination, provided a dentist studies both aspects of his occupation, and is willing to consider his patient's point of view as well as that of his own.

In all the relationships of life consideration for the other person's interests as well as one's own constitutes itself one of the chief factors in substantial success, and in a professional occupation this holds true more particularly than in any other calling. Let the dentist follow this plan in his dealing with his patients, and he will find that in the fulness of years he will reap a rich reward in pleasurable satisfaction, and in the end he will not suffer from it financially.—C. N. Johnson, *Oral Health.*

